# Complete genome sequence of *Truepera radiovictrix* type strain (RQ-24^T^)

**DOI:** 10.4056/sigs.1563919

**Published:** 2011-02-22

**Authors:** Natalia Ivanova, Christine Rohde, Christine Munk, Matt Nolan, Susan Lucas, Tijana Glavina Del Rio, Hope Tice, Shweta Deshpande, Jan-Fang Cheng, Roxane Tapia, Cliff Han, Lynne Goodwin, Sam Pitluck, Konstantinos Liolios, Konstantinos Mavromatis, Natalia Mikhailova, Amrita Pati, Amy Chen, Krishna Palaniappan, Miriam Land, Loren Hauser, Yun-Juan Chang, Cynthia D. Jeffries, Evelyne Brambilla, Manfred Rohde, Markus Göker, Brian J. Tindall, Tanja Woyke, James Bristow, Jonathan A. Eisen, Victor Markowitz, Philip Hugenholtz, Nikos C. Kyrpides, Hans-Peter Klenk, Alla Lapidus

**Affiliations:** 1 DOE Joint Genome Institute, Walnut Creek, California, USA; 2 DSMZ - German Collection of Microorganisms and Cell Cultures GmbH, Braunschweig, Germany; 3 Los Alamos National Laboratory, Bioscience Division, Los Alamos, New Mexico, USA; 4 Biological Data Management and Technology Center, Lawrence Berkeley National Laboratory, Berkeley, California, USA; 5 Oak Ridge National Laboratory, Oak Ridge, Tennessee, USA; 6 HZI – Helmholtz Centre for Infection Research, Braunschweig, Germany; 7 University of California Davis Genome Center, Davis, California, USA; 8 Australian Centre for Ecogenomics, School of Chemistry and Molecular Biosciences, The University of Queensland, Brisbane, Australia

**Keywords:** aerobic, chemoorganotrophic, non-motile, thermophilic, facultatively halophilic, alkaliphilic, radiation resistant, Gram-indeterminate, spherical-shaped, *Trueperaceae*, GEBA

## Abstract

*Truepera radiovictrix* Albuquerque *et al.* 2005 is the type species of the genus *Truepera* within the phylum “*Deinococcus/Thermus*”. *T. radiovictrix* is of special interest not only because of its isolated phylogenetic location in the order *Deinococcales*, but also because of its ability to grow under multiple extreme conditions in alkaline, moderately saline, and high temperature habitats. Of particular interest is the fact that, *T. radiovictrix* is also remarkably resistant to ionizing radiation, a feature it shares with members of the genus *Deinococcus*. This is the first completed genome sequence of a member of the family *Trueperaceae* and the fourth type strain genome sequence from a member of the order *Deinococcales*. The 3,260,398 bp long genome with its 2,994 protein-coding and 52 RNA genes consists of one circular chromosome and is a part of the *** G****enomic* *** E****ncyclopedia of* *** B****acteria and* *** A****rchaea * project.

## Introduction

Strain RQ-24^T^ (= DSM 17093 = LMG 22925 = CIP 108686) is the type strain of *Truepera radiovictrix* which is the sole and type species of the genus *Truepera* [1,2]. The generic name of strain RQ-24^T^ derives from the name “Trüper”, in honor of the German microbiologist Hans G. Trüper. The species epithet is derived from the Latin *radiovictrix*, the vanquisher of radiation [1]. Strain RQ-24^T^ was isolated in 2005 from a hot spring within a geothermal area located along an almost vertical wall and dry bed of the stream Ribeira Quente, about 500m south-east of a geothermal area on the eastern edge of the town Furnas, Azores [1]. Close to the area where RQ-24^T^ was isolated an accompanying isolate, strain TU-8, was also obtained [1]. Strains RQ-24^T^ and TU-8 share most physiological features as well as identical 16S rRNA sequence. The two *T. radiovictrix* strains also share many chemotaxonomic and physiological characteristics with the members of the genus *Deinococcus*, including the extreme resistance to ionizing radiation. Here we present a summary classification and a set of features for *T. radiovictrix* RQ-24^T^, together with the description of the complete genomic sequencing and annotation.

## Classification and features

**Figure 1:**
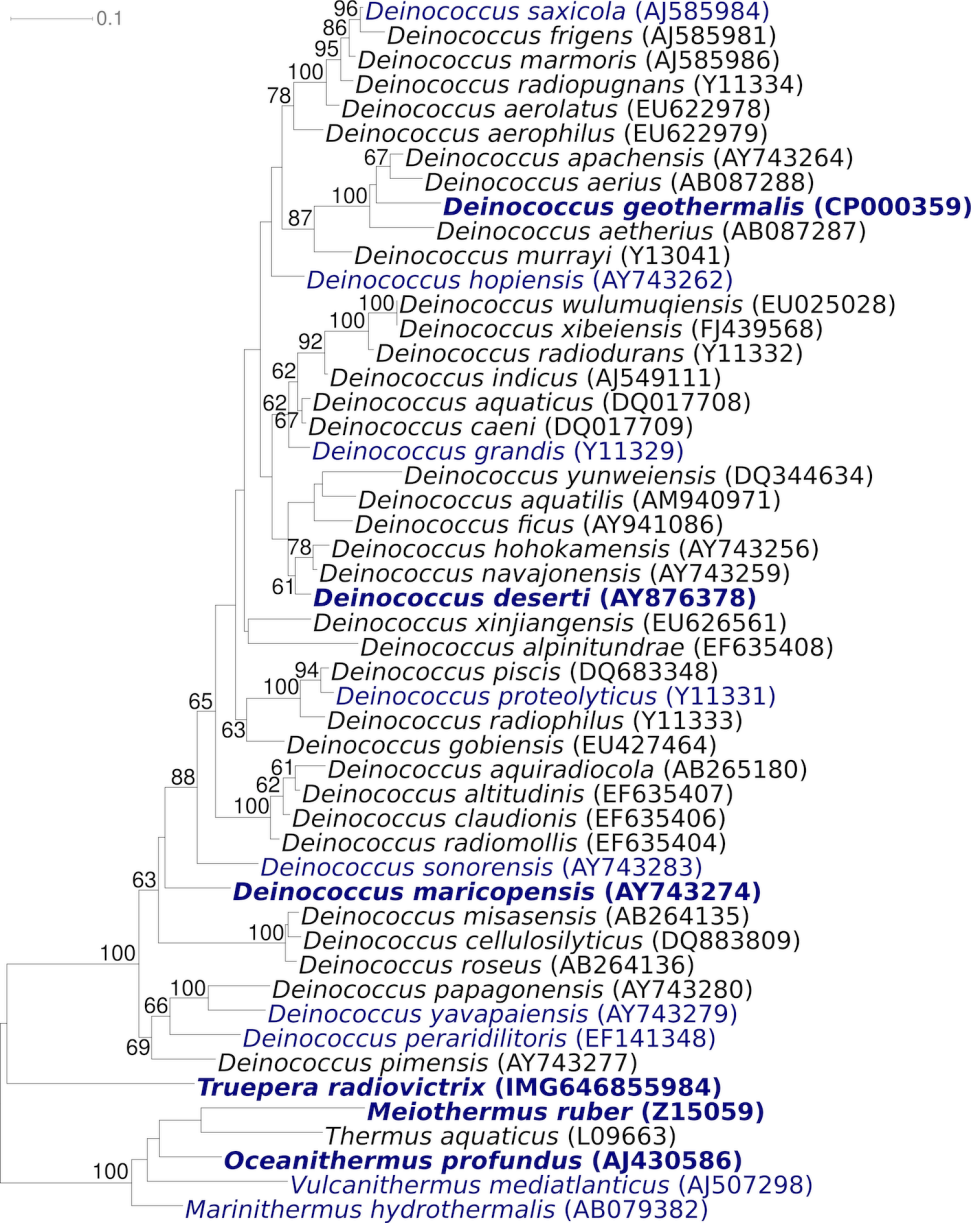
Phylogenetic tree highlighting the position of *T. radiovictrix* relative to the type strains of the other species within the class ‘*Deinococci*’. The tree was inferred from 1,457 aligned characters [3,4] of the 16S rRNA gene sequence under the maximum likelihood criterion [5] and rooted in accordance with the current taxonomy. The branches are scaled in terms of the expected number of substitutions per site. Numbers above branches are support values from 1,000 bootstrap replicates [6] if larger than 60%. Lineages with type strain genome sequencing projects registered in GOLD [7] are shown in blue, publicly available complete genome sequences [CP002361, *Oceanithermus profundus*] and published genomes [8-11] in bold. The genome of *D. radidurans* published by White *at al.* in 1999 [12] later turned out to be not from the type strain [13].

Figure 1 shows the phylogenetic neighborhood of strain RQ-24^T^ in a 16S rRNA based tree. The sequences of the two 16S rRNA gene copies in the genome differ from each other by four nucleotides, and differ by up to three nucleotides from the previously published 16S rRNA sequence (DQ022076), which contains one ambiguous base call.

A representative genomic 16S rRNA sequence of strain RQ-2^T^ was compared using NCBI BLAST under default settings (e.g., considering only the high-scoring segment pairs (HSPs) from the best 250 hits) with the most recent release of the Greengenes database [14] and the relative frequencies, weighted by BLAST scores, of taxa and keywords (reduced to their stem [15]) were determined. The five most frequent genera were *Deinococcus* (84.0%), *Truepera* (8.0%), *Oceanithermus* (6.3%), *Thiocapsa* (0.9%) and *Thiobaca* (0.8%) (98 hits in total). Regarding the two hits to sequences from members of the species, the average identity within HSPs was 99.7%, whereas the average coverage by HSPs was 97.6%. Among all other species, the one yielding the highest score was *O. profundus*, which corresponded to an identity of 87.5% and an HSP coverage of 61.9%. The highest-scoring environmental sequence was EU924247 ('Microbiology and geochemistry Little Hot Creek hot spring sediment temperature 80 degrees C clone LHC1 L4 D07'), which showed an identity of 96.6% and an HSP coverage of 91.4%. The five most frequent keywords within the labels of environmental samples which yielded hits were 'rock' (3.1%), 'microbi' (2.9%), 'skin' (2.1%), 'soil' (1.9%) and 'air' (1.6%) (152 hits in total). The five most frequent keywords within the labels of environmental samples which yielded hits of a higher score than the highest scoring species were 'rock' (4.2%), 'microbi' (2.7%), 'air' (2.4%), 'soil' (2.1%) and 'cabin/commerci' (2.0%) (60 hits in total), indicating the existence of close relatives of the strain also in less extreme habitats.

**Figure 2:**
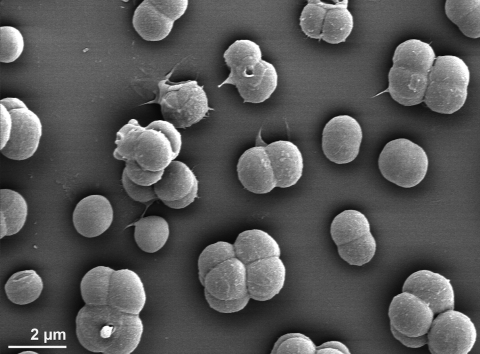
Scanning electron micrograph of *T. radiovictrix* RQ-24^T^

The non-motile, red-pigmented cells of RQ-24^T^ are spherical and approximately 1.25-2.0 µm in diameter, forming predominantly pairs or tetrads (Figure 2 and Table 1). Ultrathin sections of the cytoplasm revealed ribosomes, a fibrillar nucleoid and tubular structures of unknown nature, as well as three distinct layers of the cell wall, the innermost layer being thin, the outermost layer being as thick as 20-90 nm, both being electron dense [1]. Strain RQ-24^T^ grows best at around 50°C, but not at 20°C or 60°C, with 1.0% NaCl in *Thermus* medium or Degryse medium 162. The acceptable salinity range supporting growth is up to 6.0% NaCl. Optimum pH is 7.5-9.5, with growth detected up to pH 11.2, but no growth was detected at pH 6.0 [1]. Strains RQ-24^T^ and TU-8 are the most alkaliphilic members of the phylum “*Deinococcus/Thermus*” [1]. Both strains are oxidase and catalase positive and use a wide range of carbohydrates, organic acids or amino acids as carbon and energy sources with a respiratory metabolism (Table 1). Unexpectedly, strains RQ-24^T^ and TU-8 are capable of fermenting glucose to lactate via homolactic fermentation [1]. Also, both strains are extremely resistant to gamma irradiation, with 60% survival rate after exposure to 5.0 kGy [1]. They share this radiation resistance trait with strains from the genus *Deinococcus*, *Kineococcus radiotolerans* and the actinobacterial genus *Rubrobacter* [25].

**Table 1:** Classification and general features of *T. radiovictrix* RQ-24^T^ according to the MIGS recommendations [16].

MIGS ID	Property	Term	Evidence code
	Current classification	Domain *Bacteria*	TAS [17]
		Phylum “*Deinococcus-Thermus*”	TAS [18,19]
		Class *Deinococci*	TAS [20,21]
		Order *Deinococcales*	TAS [22]
		Family *Trueperaceae*	TAS [1,2]
		Genus *Truepera*	TAS [1,2]
		Species *Truepera radiovictrix*	TAS [1,2]
		Type strain RQ-24	TAS [1]
	Gram stain	indeterminate	TAS [1]
	Cell shape	spherical, mostly in pairs or tetrads	TAS [1]
	Motility	non-motile	TAS [1]
	Sporulation	none	TAS [1]
	Temperature range	25-55°C	TAS [1]
	Optimum temperature	50°C	TAS [1]
	Salinity	1% NaCl	TAS [1]
MIGS-22	Oxygen requirement	strictly aerobic	TAS [1]
	Carbon source	carbohydrates, organic acids, amino acids	TAS [1]
	Energy source	chemoorganotrophic	TAS [1]
MIGS-6	Habitat	hot spring	TAS [1]
MIGS-15	Biotic relationship	free-living	TAS [1]
MIGS-14	Pathogenicity	none	NAS
	Biosafety level	1	TAS [23]
	Isolation	hot spring runoff in geothermal area	TAS [1]
MIGS-4	Geographic location	River Ribeira Quente, near Furnas, Island of Sao Miguel, Azores	TAS [1]
MIGS-5	Sample collection time	2004 or before	TAS [1]
MIGS-4.1	Latitude	37.95	NAS
MIGS-4.2	Longitude	-25.49	NAS
MIGS-4.3	Depth	not reported	
MIGS-4.4	Altitude	109 meter	NAS

Evidence codes - IDA: Inferred from Direct Assay (first time in publication); TAS: Traceable Author Statement (i.e., a direct report exists in the literature); NAS: Non-traceable Author Statement (i.e., not directly observed for the living, isolated sample, but based on a generally accepted property for the species, or anecdotal evidence). These evidence codes are from of the Gene Ontology project [24]. If the evidence code is IDA, then the property was directly observed by one of the authors or an expert mentioned in the acknowledgements.

### Chemotaxonomy

All attempts to identify a peptidoglycan of strain RQ-24^T^ failed [1]. The polar lipids comprised as complex mixture of glycolipids and phospholipids, although no attempt has been made to compare them with the characteristic compounds found in members of the orders *Deinococcales* or *Thermales*. The major respiratory quinone is menaquinone 8 (MK-8). The fatty acids are predominantly saturated branched acids of which *anteiso*-C_15:0_ (38.6%), anteiso-C_17_ (17.2%) and *iso*-C_17:0_ (16.6%) as well as *iso*-C_16:0_ (6.9%). One acyl compound has an equivalent chain length (ECL) consistent with iso-C_18:0_ 1,2-diol and another compound with ECL 16.090 probably representing *iso*-C_15:0_ diol. The presence of the long-chain 1,2 diols is unknown in members of the genus *Deinococcus* (although the methods normally used would not identify them)*,* while they are found in some other members of the genera *Thermus* and *Meiothermus*. Despite the fact that members of the species *T. radiovictrix* is described as being red pigmented there is no data on the nature of the pigments.

## Genome sequencing and annotation

### Genome project history

This organism was selected for sequencing on the basis of its phylogenetic position [26], and is part of the *** G****enomic* *** E****ncyclopedia of* *** B****acteria and* *** A****rchaea * project [27]. The genome project is deposited in the Genomes OnLine Database [7] and the complete genome sequence is deposited in GenBank. Sequencing, finishing and annotation were performed by the DOE Joint Genome Institute (JGI). A summary of the project information is shown in Table 2.

**Table 2:** Genome sequencing project information

**MIGS ID**	**Property**	**Term**
MIGS-31	Finishing quality	Finished
MIGS-28	Libraries used	Three genomic libraries: one 454 pyrosequence standard library, one 454 PE library (18 kb insert size), one Illumina library
MIGS-29	Sequencing platforms	Illumina GAii, 454 GS FLX Titanium
MIGS-31.2	Sequencing coverage	70.6 × Illumina; 82.5 × pyrosequence
MIGS-30	Assemblers	Newbler version 2.1-PreRelease-4-28-2009-gcc-3.4.6-threads, Velvet, phrap
MIGS-32	Gene calling method	Prodigal 1.4, GenePRIMP
	INSDC ID	CP002049
	Genbank Date of Release	May 28, 2010
	GOLD ID	Gc01303
	NCBI project	38371
	Database: IMG-GEBA	2502957036
MIGS-13	Source material identifier	DSM 17093
	Project relevance	Tree of Life, GEBA

### Growth conditions and DNA isolation

*T. radiovictrix* RQ-24^T^, DSM 17093, was grown in DSMZ medium 1033 (*Thermus* Medium) [28] at 50°C. DNA was isolated from 0.5-1 g of cell paste using MasterPure Gram-positive DNA purification kit (Epicentre MGP04100) following the standard protocol as recommended by the manufacturer, with modification st/LALM for cell lysis as described in Wu *et al*. [27]. DNA of strain RQ-24^T^ is available through the DNA Bank Network [29,30].

### Genome sequencing and assembly

The genome was sequenced using a combination of Illumina and 454 sequencing platforms. All general aspects of library construction and sequencing can be found at the JGI website [31]. Pyrosequencing reads were assembled using the Newbler assembler version 2.1-PreRelease-4-28-2009-gcc-3.4.6-threads (Roche). The initial Newbler assembly consisting of 75 contigs in five scaffolds was converted into a phrap [32] assembly by making fake reads from the consensus, to collect the read pairs in the 454 paired end library. Illumina GAii sequencing data (230.2 Mb) was assembled with Velvet [33] and the consensus sequences were shredded into 1.5 kb overlapped fake reads and assembled together with the 454 data. The 454 draft assembly was based on 268.9 Mb 454 draft data and all of the 454 paired end data. Newbler parameters are -consed -a 50 -l 350 -g -m -ml 20. The Phred/Phrap/Consed software package [32] was used for sequence assembly and quality assessment in the subsequent finishing process. After the shotgun stage, reads were assembled with parallel phrap (High Performance Software, LLC). Possible mis-assemblies were corrected with gapResolution [31], Dupfinisher, or sequencing cloned bridging PCR fragments with subcloning or transposon bombing (Epicentre Biotechnologies, Madison, WI) [34]. Gaps between contigs were closed by editing in Consed, by PCR and by Bubble PCR primer walks (J.-F.Chang, unpublished). A total of 336 additional reactions were necessary to close gaps and to raise the quality of the finished sequence. Illumina reads were also used to correct potential base errors and increase consensus quality using a software Polisher developed at JGI [35]. The error rate of the completed genome sequence is less than 1 in 100,000. Together, the combination of the Illumina and 454 sequencing platforms provided 153.1 × coverage of the genome. The final assembly contained 736,380 pyrosequence and 6,393,275 Illumina reads.

### Genome annotation

Genes were identified using Prodigal [36] as part of the Oak Ridge National Laboratory genome annotation pipeline, followed by a round of manual curation using the JGI GenePRIMP pipeline [37]. The predicted CDSs were translated and used to search the National Center for Biotechnology Information (NCBI) nonredundant database, UniProt, TIGR-Fam, Pfam, PRIAM, KEGG, COG, and InterPro databases. Additional gene prediction analysis and functional annotation was performed within the Integrated Microbial Genomes - Expert Review (IMG-ER) platform [38].

## Genome properties

**Figure 3:**
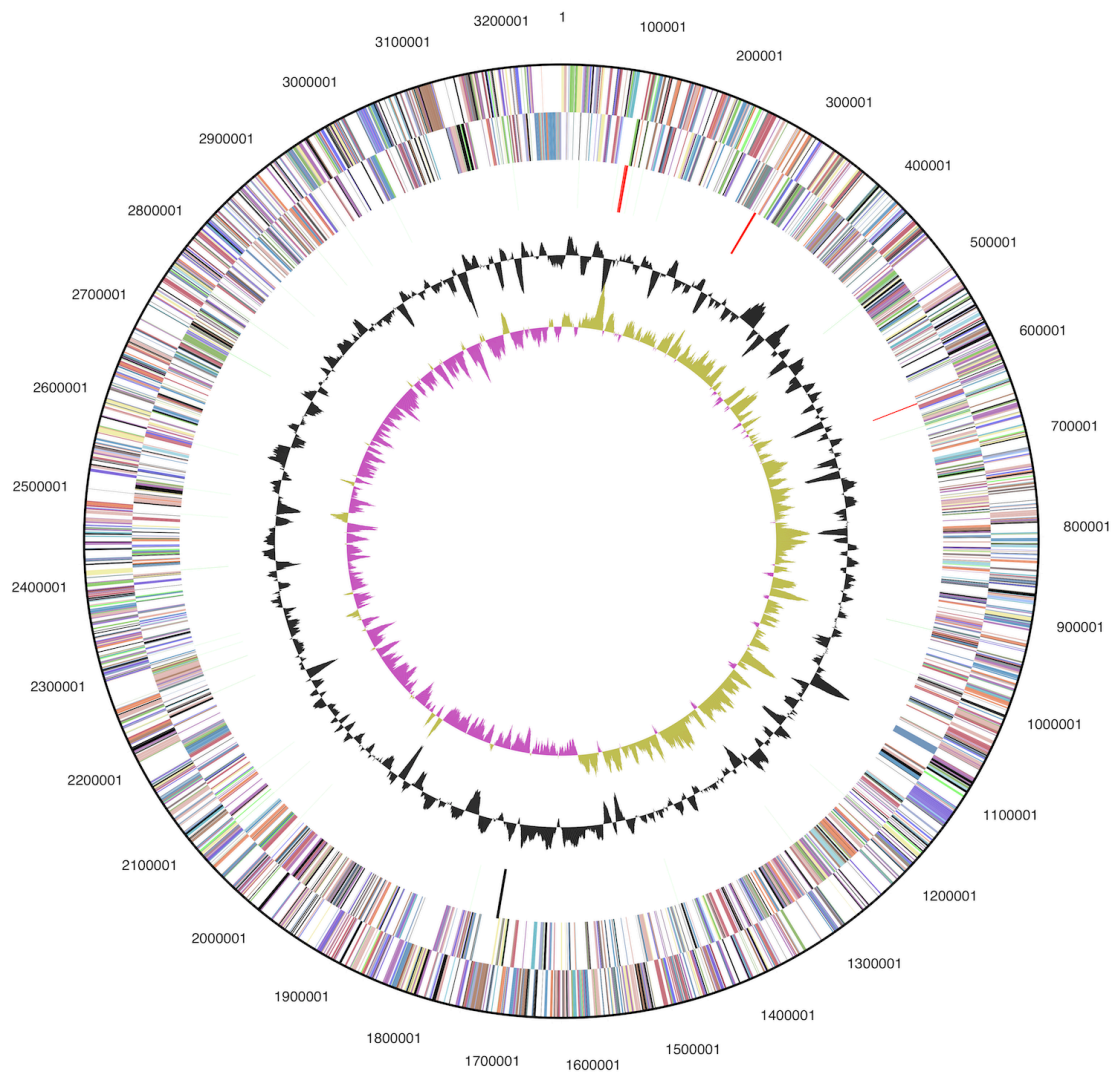
Graphical circular map of the chromosome. From outside to the center: Genes on forward strand (color by COG categories), Genes on reverse strand (color by COG categories), RNA genes (tRNAs green, rRNAs red, other RNAs black), GC content, GC skew.

The genome consists of a 3,260,398 bp long chromosome with a G+C content of 68.1% (Table 3 and Figure 3). Of the 3,046 genes predicted, 2,994 were protein-coding genes, and 52 RNAs; 49 pseudogenes were also identified. The majority of the protein-coding genes (73.4%) were assigned with a putative function while the remaining ones were annotated as hypothetical proteins. The distribution of genes into COGs functional categories is presented in Table 4.

**Table 3:** Genome Statistics

**Attribute**	**Value**	**% of Total**
Genome size (bp)	3,260,398	100.00%
DNA coding region (bp)	2,862,171	87.79%
DNA G+C content (bp)	2,221,603	68.14%
Number of replicons	1	
Extrachromosomal elements	0	
Total genes	3,046	100.00%
RNA genes	52	1.71%
rRNA operons	2	
Protein-coding genes	2,994	98.29%
Pseudo genes	49	1.61%
Genes with function prediction	2,235	73.37%
Genes in paralog clusters	370	12.15%
Genes assigned to COGs	2,272	74.59%
Genes assigned Pfam domains	2,385	78.30%
Genes with signal peptides	1,177	38.64%
Genes with transmembrane helices	709	23.28%
CRISPR repeats	9	

**Table 4:** Number of genes associated with the general COG functional categories

**Code**	**value**	**%age**	**Description**
J	149	5.9	Translation, ribosomal structure and biogenesis
A	0	0.0	RNA processing and modification
K	126	5.0	Transcription
L	135	5.4	Replication, recombination and repair
B	2	0.1	Chromatin structure and dynamics
D	29	1.2	Cell cycle control, cell division, chromosome partitioning
Y	0	0.0	Nuclear structure
V	40	1.6	Defense mechanisms
T	93	3.7	Signal transduction mechanisms
M	128	5.1	Cell wall/membrane/envelope biogenesis
N	14	0.6	Cell motility
Z	0	0.0	Cytoskeleton
W	0	0.0	Extracellular structures
U	34	1.4	Intracellular trafficking, secretion, and vesicular transport
O	82	3.3	Posttranslational modification, protein turnover, chaperones
C	148	5.9	Energy production and conversion
G	244	9.7	Carbohydrate transport and metabolism
E	271	10.7	Amino acid transport and metabolism
F	79	3.1	Nucleotide transport and metabolism
H	104	4.1	Coenzyme transport and metabolism
I	84	3.3	Lipid transport and metabolism
P	170	6.7	Inorganic ion transport and metabolism
Q	63	2.5	Secondary metabolites biosynthesis, transport and catabolism
R	343	13.6	General function prediction only
S	187	7.4	Function unknown
-	774	25.4	Not in COGs

## References

[1] Albuquerque L Simoes C Nobre MF Pino NM Battista JR Silva MT Rainey FA Da Costa MS *Truepera radiovictrix* gen. nov., sp. nov., a new radiation resistant species and the proposal of *Trueperaceae* fam. nov. FEMS Microbiol Lett 2005; 247:161-169 10.1016/j.femsle.2005.05.002 15927420

[2] Euzéby J Validation of publication of new names and new combinations previously effectively published outside the IJSEM. Int J Syst Evol Microbiol 2005; 55:1743-1745 10.1099/ijs.0.63996-0 16166658

[3] Castresana J Selection of conserved blocks from multiple alignments for their use in phylogenetic analysis. Mol Biol Evol 2000; 17:540-552 10742046 10.1093/oxfordjournals.molbev.a026334

[4] Lee C Grasso C Sharlow MF Multiple sequence alignment using partial order graphs. Bioinformatics 2002; 18:452-464 10.1093/bioinformatics/18.3.452 11934745

[5] Stamatakis A Hoover P Rougemont J A rapid bootstrap algorithm for the RAxML Web servers. Syst Biol 2008; 57:758-771 10.1080/10635150802429642 18853362

[6] Pattengale ND Alipour M Bininda-Emonds ORP Moret BME Stamatakis A How many bootstrap replicates are necessary? Lect Notes Comput Sci 2009; 5541:184-200 10.1007/978-3-642-02008-7_13 20377449

[7] Liolios K Chen IM Mavromatis K Tavernarakis N Hugenholtz P Markowitz VM Kyrpides NC The Genomes On Line Database (GOLD) in 2009: status of genomic and metagenomic projects and their associated metadata. Nucleic Acids Res 2010; 38:D346-D354 10.1093/nar/gkp848 19914934 PMC2808860

[8] de Groot A Dulermo R Ortet P Blanchard L Guerin P Fernandez B Vacherie B Dossat C Jolivet E Siguier P Alliance of proteomics and genomics to unravel the specificities of Sahara bacterium *Deinococcus deserti.* PLoS Genet 2009; 5:e1000434 10.1371/journal.pgen.1000434 19370165 PMC2669436

[9] Makarova KS Omelchenko MV Gaidamakova EK Matrosova VY Vasilenko A Zhai M Lapidus A Copeland A Kim E Land M *Deinococcus geothermalis*: the pool of extreme radiation resistance genes shrinks. PLoS ONE 2007; 2:e955 10.1371/journal.pone.0000955 17895995 PMC1978522

[10] Pukall R Zeytun A Lucas A Lapidus A Hammon N Deshpande S Nolan M Cheng JF Pitluck S Liolios K Complete genome sequence of *Deinococcus maricopensis* type strain (LB-34^T^). Stand Genomic Sci 2011; (Next issue). 10.4056/sigs.1633949 PMC3111983 21677853

[11] Tindall BJ Sikorski J Lucas S Goltsman E Copeland A Glavina Del Rio T Nolan M Tice H Cheng JF Han C Complete genome sequence of *Meiothermus ruber* type strain (21^T^). Stand Genomic Sci 2010; 3:26-36 10.4056/sigs.1032748 21304689 PMC3035268

[12] White O Eisen JA Heidelberg JF Hickey EK Peterson JD Dodson RJ Haft DH Gwinn ML Nelson WC Richardson DL Genome sequence of the radioresistant bacterium *Deinococcus radiodurans* R1. Science 1999; 286:1571-1577 10.1126/science.286.5444.1571 10567266 PMC4147723

[13] White O Eisen JA Heidelberg JF Hickey EK Peterson JD Dodson RJ Haft DH Gwinn ML Nelson WC Richardson DL Erratum: Genome sequence of the radioresistant bacterium *Deinococcus radiodurans* R1. Science 2004; 303:1571-1577 10.1126/science.286.5444.1571 PMC4147723 10567266

[14] DeSantis TZ Hugenholtz P Larsen N Rojas M Brodie EL Keller K Huber T Dalevi D Hu P Andersen GL Greengenes, a Chimera-checked 16S rRNA Gene Database and Workbench Compatible with ARB. Appl Environ Microbiol 2006; 72:5069-5072 10.1128/AEM.03006-05 16820507 PMC1489311

[15] Porter MF An algorithm for suffix stripping. Program: electronic library and information systems 1980; **14**:130-137. 10.1108/eb046814

[16] Field D Garrity G Gray T Morrison N Selengut J Sterk P Tatusova T Thomson N Allen MJ Angiuoli SV The minimum information about a genome sequence (MIGS) specification. Nat Biotechnol 2008; 26:541-547 10.1038/nbt1360 18464787 PMC2409278

[17] Woese CR Kandler O Wheelis ML Towards a natural system of organisms: proposal for the domains *Archaea, Bacteria*, and *Eucarya.* Proc Natl Acad Sci USA 1990; 87:4576-4579 10.1073/pnas.87.12.4576 2112744 PMC54159

[18] Weisburg WG Giovannoni SJ Woese CR The *Deinococcus-Thermus* phylum and the effect of rRNA composition on phylogenetic tree construction. Syst Appl Microbiol 1989; 11:128-134 11542160 10.1016/s0723-2020(89)80051-7

[19] Garrity GM, Holt JG. 2001. Taxonomic outline of the *Archaea* and *Bacteria*, p. 155-166. *In* G. M. Garrity, D. R. Boone, and R. W. Castenholz (ed.), Bergey's Manual of Systematic Bacteriology, 2nd ed, vol. 1. Springer, New York.

[20] List Editor Validation List no. 85. Validation of publication of new names and new combinations previously effectively published outside the IJSEM. Int J Syst Evol Microbiol 2002; 52:685-690 10.1099/ijs.0.02358-0 12054225

[21] Garrity GM, Holt JG. Class I. *Deinococci* class. nov. In: Garrity GM, Boone DR, Castenholz RW (eds), Bergey's Manual of Systematic Bacteriology, Second Edition, Volume 1, Springer, New York, 2001, p. 395.

[22] Rainey FA Nobre MF Schumann P Stackebrandt E da Costa MS Phylogenetic diversity of the deinococci as determined by 16S ribosomal DNA sequence comparison. Int J Syst Bacteriol 1997; 47:510-514 10.1099/00207713-47-2-510 9103641

[23] Classification of bacteria and archaea in risk groups. http://www.baua.de TRBA 466.

[24] Ashburner M Ball CA Blake JA Botstein D Butler H Cherry JM Davis AP Dolinski K Dwight SS Eppig JT Gene Ontology: tool for the unification of biology. Nat Genet 2000; 25:25-29 10.1038/75556 10802651 PMC3037419

[25] Ferreira AC Nobre MF Moore E Rainey FA Battista JR da Costa MS Characterization and radiation resistance of new isolates of *Rubrobacter radiotolerans* and *Rubrobacter xylanophilus.* Extremophiles 1999; 3:235-238 10.1007/s007920050121 10591012

[26] Klenk HP Goeker M En route to a genome-based classification of *Archaea* and *Bacteria*? Syst Appl Microbiol 2010; 33:175-182 10.1016/j.syapm.2010.03.003 20409658

[27] Wu D Hugenholtz P Mavromatis K Pukall R Dalin E Ivanova NN Kunin V Goodwin L Wu M Tindall BJ A phylogeny-driven genomic encyclopaedia of Bacteria and Archaea. Nature 2009; 462:1056-1060 10.1038/nature08656 20033048 PMC3073058

[28] List of growth media used at DSMZ: http//www.dsmz.de/microorganisms/media_list.php

[29] Gemeinholzer B Dröge G Zetzsche H Haszprunar G Klenk HP Güntsch A Berendsohn WG Wägele JW The DNA Bank Network: the start from a German initiative. Biopreservation and Biobanking (In press). 10.1089/bio.2010.0029 24850206

[30] DNA bank Network http://www.dnabank-network.org

[31] The DOE Joint Genome Institute http://www.jgi.doe.gov

[32] Phrap and Phred for Windows, MacOS, Linux, and Unix. http://www.phrap.com

[33] Zerbino DR Birney E Velvet: algorithms for de novo short read assembly using de Bruijn graphs. Genome Res 2008; 18:821-829 10.1101/gr.074492.107 18349386 PMC2336801

[34] Sims D Brettin T Detter JC Han C Lapidus A Copeland A Glavina Del Rio T Nolan M Chen F Lucas S Complete genome sequence of *Kytococcus sedentarius* type strain (541^T^). Stand Genomic Sci 2009; 1:12-20 10.4056/sigs.761 21304632 PMC3035214

[35] Lapidus A, LaButti K, Foster B, Lowry S, Trong S, Goltsman E. POLISHER: An effective tool for using ultra short reads in microbial genome assembly and finishing. AGBT, Marco Island, FL, 2008.

[36] Hyatt D Chen GL LoCascio PF Land ML Larimer FW Hauser LJ Prodigal: prokaryotic gene recognition and translation initiation site identification. BMC Bioinformatics 2010; 11:119 10.1186/1471-2105-11-119 20211023 PMC2848648

[37] Pati A Ivanova NN Mikhailova N Ovchinnikova G Hooper SD Lykidis A Kyrpides NC GenePRIMP: a gene prediction improvement pipeline for prokaryotic genomes. Nat Methods 2010; 7:455-457 10.1038/nmeth.1457 20436475

[38] Markowitz VM Ivanova NN Chen IMA Chu K Kyrpides NC IMG ER: a system for microbial genome annotation expert review and curation. Bioinformatics 2009; 25:2271-2278 10.1093/bioinformatics/btp393 19561336

